# An Exercise Prescription for COVID-19 Pandemic

**DOI:** 10.12669/pjms.36.7.2929

**Published:** 2020

**Authors:** Zait Burak Aktug, Nazlım Aktug Demir

**Affiliations:** 1 Dr. Zait Burak Aktuğ Niğde Ömer Halisdemir University, Sports Science Faculty, Nigde, Turkey; 2 Dr. Nazlım Aktuğ Demir Selcuk University, Faculty of Medicine, Department of Clinical Microbiology and Infectious Diseases, Konya, Turkey

**Keywords:** COVID-19, Exercise prescription, Health

## Abstract

COVID-19 is an alarming public health concern worldwide. COVID-19 is highly contagious and has no approved treatment or vaccine yet. Therefore, the best strategy is prevention. Studies have shown that a healthy lifestyle, regular exercise, balanced eating, and quality sleep are the key elements for protection from this disease. We are going through a distressful period as a nation and as the human race in general. We need to manage this period in the best way possible in physiological and psychological terms. Physical activity is one of the major steps in managing this period in a healthy way. Individuals should be provided information about exercise so that they can perform correct physical activities within their means. This article presents an exercise prescription that can be followed in the days of the COVID-19 pandemic.

## INTRODUCTION

COVID-19 is an alarming public health concern worldwide. The clinical findings of COVID-19 infection exhibit a broad spectrum of conditions ranging from asymptomatic disease or mild upper respiratory tract infection to severe viral pneumonia involving respiratory failure that can result in death.^1^,^2^

Coronaviruses, in which SARS-CoV-2 is a member, are a large virus family at times causing only self-limiting mild infections widely seen in the community such as common cold and at other times leading to more serious infections such as Middle East Respiratory Syndrome (MERS) and Severe Acute Respiratory Syndrome (SARS). SARS-CoV made its appearance in 2003 as a previously unknown virus and caused deaths of a large number of people. MERS-CoV was defined first approximately 10 years later in September 2012 in Saudi Arabia. Finally, in December 2019, the SARS-CoV-2 agent was defined in the pneumonia outbreak that took place in China with the city of Wuhan as the centre. This new disease was named COVID-19.^3^,^4^ COVID-19 is highly contagious and has no approved treatment or vaccine yet. Therefore, the best strategy is protection. Studies have shown that a healthy lifestyle, regular exercise, balanced eating, and quality sleep are the key elements for protection from this disease.^5^,^6^

Isolation is important to control the disease, but it also involves many unfavourable physiological and psychological effects.^7^ Changing lifestyles and behaviours in this period may result in inadequate physical activity and movement,^8^,^9^ which will pave the way for diabetes, hypertension, cardiovascular diseases, respiratory diseases, and mental disorders.^9^,^10^ For individuals to go through this process in a healthy manner, they will need to adapt to this kind of living. It is essential to remain active and maintain an exercise routine during quarantine periods to protect mental and physical health.^11^

### Exercise and Health

The World Health Organization has defined health as a state of complete physical, mental, and social well-being and not merely the absence of infirmity or disease. Health is an inseparable whole with all its physical and mental dimensions and affects each other.^12^ Uncertainty about the time it will take to end the COVID-19 pandemic, fear and anxiety arousing news in the social media, quarantine processes, etc. lead to a variety of problems including stress, anxiety, depression, and sleep disorders.^13^,^14^

Exercises are planned and repetitive movements structured to protect, improve, and develop physical health. Regular exercise have major contributions to the prevention and treatment of some health problems including diabetes, obesity, hyperlipidemia, cardiovascular diseases, some cancer types, and osteoporosis.^15^-^21^ Exercise also has other positive effects such as reducing anxiety, stress and depression, sustaining mental health, and achieving psychological well-being.^22^,^23^ Exercise is known to enhance self-perception, promote social functioning, and improve night sleep by limiting daytime sleep.^22^,^24^-^26^

The effects of exercise on mental health occur in two ways: The first one is associated with the increase in synaptic transmission of monoamines induced by physical activity, which resembles the mechanism of action of antidepressant drugs.^27^,^28^ The second occurs when physical activity causes the secretion of endorphin (primarily beta-endorphin), which suppresses the central nervous system creating a state of calmness and mental relaxation.^29^,^30^

Studies investigating the effects of exercise on mental health in the literature use various exercise types with varying intensities, frequencies, and durations. There are different opinions as to which types of exercise are more effective on mental health.^31^-^34^

Two points should be considered when deciding on the exercises to be carried out during the COVID-19 pandemic. It is important to choose the exercises that will simultaneously strengthen both our mental health and immune system, the latter being our core protector in this time of disease.

Many people are unwilling to engage in physical activity due to working conditions, children, spouse, insufficient time, physical constraints, boring nature of some exercises, and sedentary lifestyle.^35^ The important thing here is to choose exercises which we would enjoy doing and will keep us occupied with the activity. Most of the patients with depression have stated that they enjoy moderate-intensity exercises (maximum heart rate 60-80%) rather than high-intensity exercises.^36^ In attempts to use high-intensity exercises as a start, approximately half of the patients choose to not continue exercise.^37^ In such cases, starting with low-intensity aerobic exercises is recommended to ensure the continuation of the exercise because they are found more enjoyable.^35^ Many studies have shown that aerobic exercises have no negative effect on mental diseases; on the contrary, they are effective in the prevention and treatment of mental diseases.^31^,^32^ High-intensity exercises are known to exert a suppressive effect on the immune system, whereas low and moderate-intensity aerobic exercises improve the immune system. Aerobic and low-intensity resistance training are argued to be more effective than aerobic exercises alone^38^,^39^ and for this reason, strength exercises alongside aerobic exercises are recommended during the COVID-19 pandemic. Since frequent workouts have been found more effective in reducing depression symptoms,^40^ efforts should be made to encourage patients to do workouts as often as possible in this period. Based on the above information, we think that practicing the exercise programs given below will yield positive results in this period.

## COVID-19 PROCESS AND EXERCISE PRESCRIPTION

* Those newly starting exercise should practice these exercises at least three days a week and those regularly exercise at least five days a week.
* Resistance exercise days should be scheduled to be between aerobic exercise days.
* Care should be taken about the intensity of exercises; excessively tiring and anaerobic workouts should be avoided.
* Those newly starting exercise should increase first the frequency, then the duration, and then the intensity of the exercise in time.

Table-I shows the intensity and duration of the exercise are between certain intervals. If you increase the intensity, you should decrease the duration. This means that intensity and duration should be inversely proportional.

**Table-I:** Exercise program for those who can train in the open air and have the equipment for strength exercises at home.

		Exercise type	Exercise Duration	Exercise Frequency	Exercise Intensity
Aerobic Exercises	Individuals who will newly start exercise	Walking (90-100 steps/minutes.)	20-35 minutes	2 days/week	MHRR 30-55%
		Cycling (12-14 km/hour)			
	Regularly exercise individuals	Walking (>120 steps/minutes.)	35-60 minutes	3-4 days/week	MHRR 60-80%
		Cycling (15-18 km/hour)			
		Jogging or running (At least 40 pulses more than resting HR)			

		*Number of Sets*	*Number of Repetitions*	*Exercise Frequency*	*Exercise Intensity*

Resistance Exercises	Individuals who will exercise for the first time	For each movement	8-12	1-2 days/week	1 RM 30-50%
		1-2 sets			
	Regularly exercise individuals	For each movement	8-12	2-3 days/week	1 RM 50-70%
		2-3 sets			

MHRR: Maximal heart rate reserve RM: Maximal repetition HR: Heart rate

**Table-II:** Exercise program for those who have to train at home.

		Exercise Type	Exercise Duration	Exercise Set	Exercise Frequency
Aerobic Exercises	Individuals newly starting exercise	a,b,e	10-20 minutes.	1-2	2 days/week
	Regularly exercise individuals	c,d,e	15-30 minutes.	2-3	3-4 days/week

		*Exercise Type*	*Number of Repetition*	*Exercise Set*	*Exercise Frequency*

Resistance Exercises	Individuals newly starting exercise	a,b,c,d,e,f	10-15	1-2	1-2 days/week
	Regularly exercise individuals	a,b,c,d,e,f	>20	2-3	2-3 days/week

* Resistance exercises should be composed of at least five movements that work out different muscle groups. These movements should involve both the lower and upper extremities. The movements should address to large and small muscle groups.
  1. *The Sample program*, 1^st^ move: squat; 2^nd^ move: shoulder press; 3^rd^ move: sit-up; 4^th^ move: leg curl; 5^th^ move: triceps extension; 6^th^ move: lat pulldown.
  2. *The Sample program*, 1^st^ move: leg extension; 2^nd^ move: barbell curl; 3^rd^ move: chin up; 4^th^ move: leg curl; 5^th^ move: bench press; 6^th^ move: shoulder press.
  3. *The Sample program*, 1^st^ move: Romanian deadlift; 2^nd^ move: z bar barbell curl; 3^rd^ move: dumbell fly; 4^th^ move: hack squat; 5^th^ move: side lateral raise; 6^th^ move: one arm dumbell row
  4. *The Sample program*, 1^st^ move: leg press; 2^nd^ move: cable push down; 3^rd^ move: sit-up; 4^th^ move: leg curl; 5^th^ move: barbell incline press; 6^th^ move: hyperextension.
* Each of these sample programs is composed of 6 movements that work out a different muscle group. The movements are designed to work out both the lower and upper extremities.
* Exercise types are shown in letters. One of the exercise types shown for aerobic exercises will be chosen.
* Muscle groups in resistance exercises are shown in letters. One movement for each muscle group will be chosen; six movements in total will be included.
* Care should be taken about the intensity of exercises; excessively tiring and anaerobic workouts should be avoided.
* Those newly starting exercise should increase first the frequency, then the duration, and then the intensity of the exercise in time.
* Resistance exercise days should be scheduled to be between aerobic exercise days.
* The intensity and duration of the exercise should be inversely proportional.

### Aerobic Exercises:

* (a) Stepping up and down on a 30-40 cm raised platform with a pace 60-70 steps/minutes.
* (b) Walking where you are with a pace 70-90 steps/minutes. (The feet should be raised a little higher than in normal walking position)
* (c) Stepping up and down on a 30-40 cm raised platform with a pace 90-110 steps/minutes.
* (d) Walking where you are with a pace > 100-120 steps/minutes. (The feet should be raised a little higher than in normal walking position)
* (e) Zumba, oriental, dance

### Resistance Exercises:

* Resistance bands were chosen when preparing the program as they are low-cost and readily available items.
* New beginners should use moderate-level resistance bands (yellow or red / moderate-level).
* Regularly exercise individuals should use high-level resistance bands (blue or black / difficult-level).

### Resistance Exercise Movements Using Resistance Bands:

* (a) Arm exercises: Biceps curl, one arm upright; triceps extension
* (b) Shoulder exercises: Lateral rise; upright row; front rise; shoulder diagonal flexion
* (c) Back exercises: Lat pulldown back; scapular retraction; band seated row
* (d) Chest exercises: Chest fly; chest press; push-up
* (e) Leg exercises: Lunge; leg press; hip adduction; hip abduction; squat
* (f) Abdominutesal exercises: Abdominutesal crunch; flutter kick; air bike crunches; knees up crunch

## CONCLUSION

We are going through a distressful period as a nation and as the human race in general. We need to manage this period in the best way possible in physiological and psychological terms. Physical activity is one of the major steps in managing this period in a healthy way. Individuals should be provided information about exercise so that they can perform correct physical activities within their means. This article presents an exercise prescription that can be followed in the days of the COVID-19 pandemic.

### Authors’ Contribution:

**ZBA:** Conceived, did manuscript writing and editing of manuscript

**NAD:** Did manuscript writing and final approval of manuscript, literature review
